# Multistaged Hybrid Percutaneous Coronary Intervention in ST-Segment Elevation Myocardial Infarction With Multivessel Disease

**DOI:** 10.1016/j.jaccas.2024.103028

**Published:** 2025-03-05

**Authors:** Belén Jiménez-Azzaoui, Pilar Jiménez-Quevedo, Nieves Gonzalo, Fernando Macaya-Ten

**Affiliations:** a Cardiology Department, Hospital Severo Ochoa, Leganés, Madrid, Spain; b Cardiology Department, Hospital Clínico San Carlos, Madrid, Spain

**Keywords:** drug-coated balloon, myocardial infarction, percutaneous coronary angiography, percutaneous coronary intervention

## Abstract

This paper presents a case of acute myocardial infarction in a patient with a thrombotic occlusion of a diffusely diseased left anterior descending artery, who also had nonculprit severe lesions in the obtuse marginal and right coronary arteries. Considering the extent of the disease and the clinical presentation, a carefully planned multistaged hybrid approach involving drug-eluting balloons and stents was chosen as the optimal revascularization strategy for this patient.

## Graphical Abstract

**Figure:**
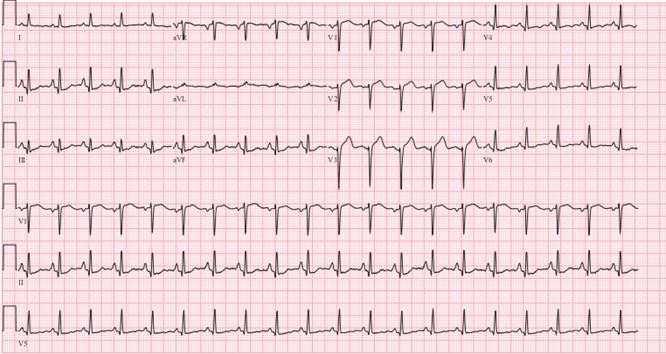

## History of Presentation

A 64-year-old woman with a history of arterial hypertension presented to the hospital reporting exertional chest pain radiating to the jaw and neck since the previous day.

**Take-Home Messages**

• This case underscores the complexities involved in managing acute myocardial infarction with concomitant multivessel coronary disease.
• The use of a combination of DBC and DES enables complete revascularization in patients with extensive coronary disease while minimizing the permanent metallic burden.

## Past Medical History

The patient had no previous medical history.

## Differential Diagnosis

The initial differential diagnoses included chronic or acute coronary syndrome, pericarditis, pulmonary embolism, and aortic dissection.

## Investigations

**Figure 1:**
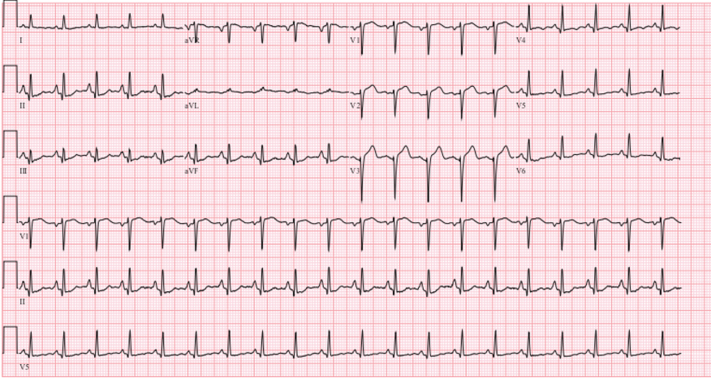
Electrocardiogram 12-lead electrocardiogram obtained at presentation demonstrating ST-segment elevation in leads V_1_ to V_3_.

Electrocardiography showed ST-segment elevation in leads V_1_ to V_3_ (Figure 1). Laboratory examination demonstrated an elevated troponin T concentration of 2,064 pg/mL (reference range: ≤16 ng/L). Transthoracic echocardiography exhibited apical hypokinesis with a preserved left ventricular ejection fraction.

## Management

**Figure 2:**
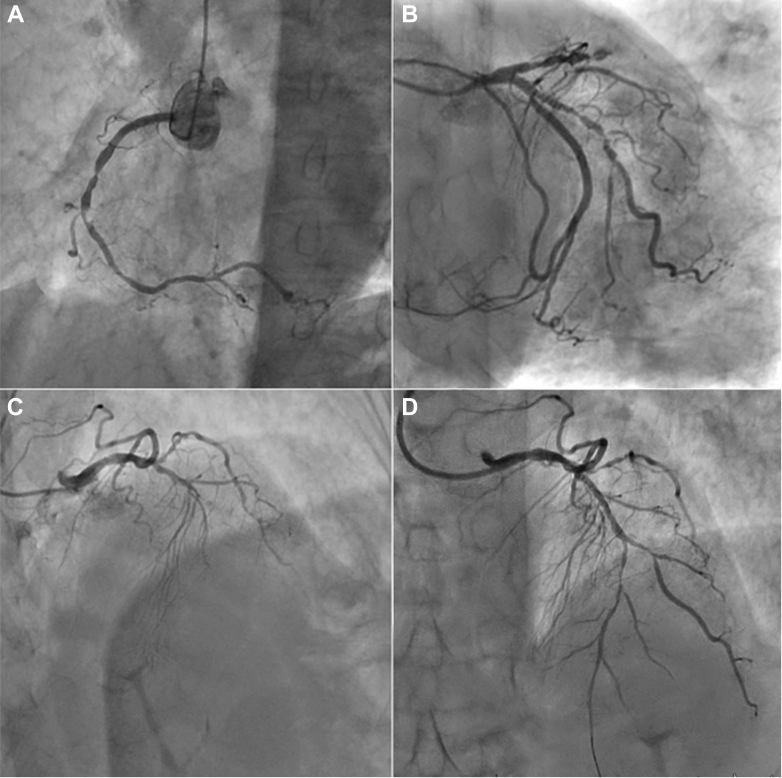
Coronary Angiography (A) Tandem severe stenoses in the mid-segment of right coronary artery. (B) Tandem severe stenoses in the proximal and middle segment of the obtuse marginal branch. (C) Left anterior descending artery (LAD) complete occlusion in right anterior oblique cranial projection. (D) Flow restoration demonstrates extensive disease into mid-distal segments of the LAD involving an important diagonal branch.

Given the clinical presentation and the complementary tests, we decided to perform an urgent coronary angiography. It was conducted following standard recommendations (radial approach and a 6-F sheath). The assessment (Figure 2, Video 1) unveiled the following findings: a proximal diffusely diseased left anterior descending artery (LAD) with total occlusion in the transition to the middle segment, severe tandem lesions in the mid-segment of the first obtuse marginal (OM) branch, and a severe stenosis in the middle segment of the right coronary artery (RCA).

Based on the presentation, the mid-LAD occlusion was considered responsible for the myocardial infarction and targeted for immediate revascularization with percutaneous coronary intervention (PCI).

**Figure 3:**
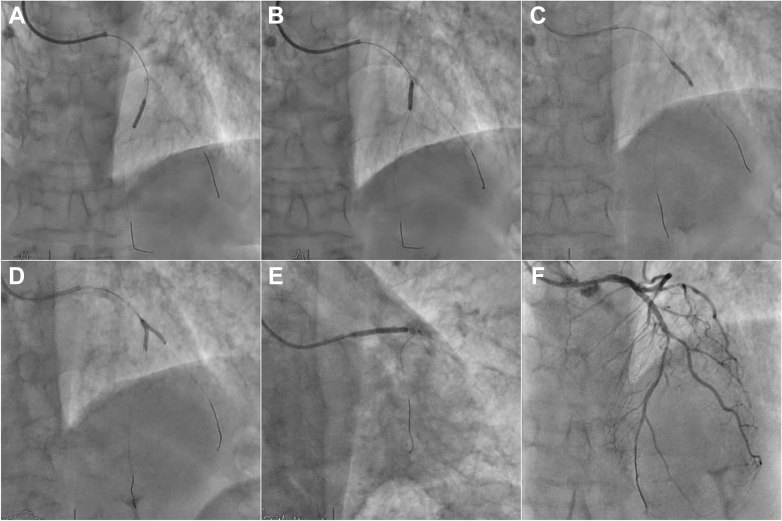
LAD Angioplasty (A) Noncompliant balloon dilation in mid-distal left anterior descending artery (LAD) stenosis. (B) Noncompliant balloon dilation in mid-LAD stenosis at bifurcation. (C) Noncompliant balloon dilation in the proximal diagonal branch at bifurcation. (D) Kissing balloon inflation technique with drug-coated balloon. (E) Drug-eluting stent implantation in the proximal LAD segment. (F) Final angiography result.

Epicardial flow was restored after gentle balloon dilation, noting that the stenosis extended down to the bifurcation with a first diagonal branch of significant size and throughout the entire mid-distal LAD (Figure 2D). As shown in Figure 3 and Video 2, several high-pressure dilations with 2.5-mm noncompliant balloons were undertaken to treat the diseased segments of mid-distal LAD and diagonal branch. After ensuring adequate lumen gain and flow, 3 paclitaxel-coated balloons dilations (2.5 × 15 mm) were used to deliver drug in the diseased and dilated segments, including a kissing-balloon dilation for the bifurcation (Figure 3D). Finally, we successfully deployed a drug-eluting stent (DES) 3 × 30 mm in the proximal LAD segment obtaining a good angiographic result (Figures 3E and 3F).

## Discussion

Advances in coronary stenting technology over the years have transformed PCI into a highly safe and effective treatment, even for patients with multivessel disease. However, the drawbacks of stenting coronary arteries become more important as the complexity and extension of the disease increases.^1^

Drug-coated balloons (DCBs) are devices coated with cytotoxic chemotherapeutic agents (eg, paclitaxel) that have become attractive because they fulfill the concept of leaving nothing behind by combining the benefits of local drug delivery without the complications of a permanent stent implantation, which allows positive vascular remodeling and restoration of vasomotor function.^2^ They are recommended in the guidelines for the treatment of in-stent restenosis.^3^ However, recent randomized clinical data have demonstrated a good efficacy and safety profile in de novo small disease.^4^ Because of this, the International DCB Consensus Group^2^ is updating its previous recommendations.

Bifurcation lesions, which account for 15% to 20% of all PCIs, present unique challenges that result in higher procedural complexities, increased restenosis rates, and poorer clinical outcomes compared with nonbifurcation cases. A hybrid strategy with DCB and DES in true bifurcations may be a sensible option to reduce target lesion failure.^5^ In cases of extensive and diffuse coronary artery disease, a hybrid approach can effectively minimize the necessary stent length, which is a recognized predictor of in-stent restenosis. Moreover, by reducing the extent of metallic implantation, this strategy may also help preserve the vessel’s natural vasomotion response and decrease the risk of neoatherosclerosis.^6^

The presented case illustrates a variety of lesion features that are eligible for treatment with DCBs: small vessel size and diffuse disease consisting in long segments affected and true bifurcations. A strategy based in the combination of DCB and DES reduces the total burden of the permanent metallic prosthesis, which may translate into less target lesion failure during follow-up. This approach also avoided stenting the mid-LAD, which permits grafting in the eventual future necessity of it.^7^ In the hybrid approach for diffuse disease, the DES is usually implanted in the proximal lesions (or main branch) and DCBs are used in more distal segments (or side branch), providing a good angiographic result of balloon dilation in the latter lesions. In this case, the DES was also implanted in the proximal LAD segment because it was the culprit lesion with ruptured and thrombotic plaque, for which there is less evidence supporting the use of DCBs. To date, only small observational studies have proven the feasibility of this approach in the treatment of diffuse coronary artery disease, underscoring the need for further prospective investigations.

## Follow-Up

**Figure 4:**
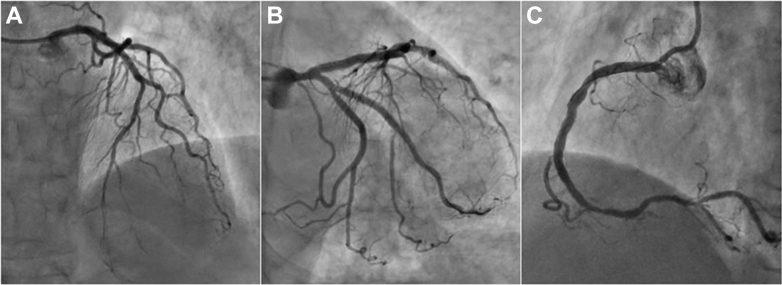
Final Angiographic Result (A) Sustained outcome of the left anterior descending artery stenosis previously treated with a hybrid strategy using a drug-eluting stent (DES) and drug-coated balloon. (B) Successful result of the right coronary artery (RCA) and obtuse marginal stenosis treated with a DES. (C) Successful result of the RCA treated with a DES.

The patient remained hospitalized with heart failure without experiencing new episodes of chest pain. During the hospitalization, a subsequent angioplasty was performed to address the stenosis in the proximal segment of the RCA using a DES (Figure 4C, Video 3). One month after discharge, another angioplasty was performed to treat the stenosis in the mid-segment of the OM (Figure 4B, Video 3). During this procedure, we observed a favorable angiography outcome of the previous angioplasties with successful outcome of the LAD stenosis treated with a hybrid strategy using DES and DCB (Figure 4A, Video 3).

## Conclusions

Managing extensive coronary disease with bifurcation and small vessel lesions through PCI presents challenges. This case depicted the successful treatment of this scenario with a multistage hybrid strategy using DES and DCB interventions. The emerging use of DCBs for complex diffuse disease brings hope in improving procedural outcomes and reducing complications. However, further research through randomized controlled trials is needed to clarify their role, especially compared with the latest generation of DESs.

## Funding Support and Author Disclosures

The authors have reported that they have no relationships relevant to the contents of this paper to disclose.

## Appendix

Video 1 Baseline diagnostic angiogram. Severe stenoses in the middle segment of the right coronary artery, severe tandem lesions in the mid-segment of the first obtuse marginal branch, and severe stenosis in the proximal left anterior descending artery segment identified as the culprit lesion with ruptured plaque

Video 2 Left Anterior Descending Artery Revascularization Using a Drug-Coated Balloon and Drug-Eluting Stent

Video 3 Angioplasty to Right Coronary Artery and Obtuse Marginal Stenosis Using a Drug-Eluting Stent Sustained outcome of the left anterior descending artery stenosis previously treated with a hybrid strategy using a drug-eluting stent and drug-coated balloon
